# The relationship between staying at home during the pandemic and the number of conceptions: A national panel data analysis

**DOI:** 10.1371/journal.pone.0289604

**Published:** 2023-08-11

**Authors:** Luca Moreno-Louzada, Naercio Menezes-Filho

**Affiliations:** 1 University of São Paulo, São Paulo, SP, Brazil; 2 Insper, São Paulo, SP, Brazil; Uniformed Services University: Uniformed Services University of the Health Sciences, UNITED STATES

## Abstract

hether the COVID-19 pandemic has changed fertility patterns is still an open question, as social isolation for long periods can impact the number of conceptions in many ways. We combine administrative data on all recent births in Brazil with daily data on individual location to estimate the relationship between the share of individuals staying close to their homes in each week and the number of conceptions in that same week, comparing municipalities with different social isolation patterns during the first semester of 2020. We find that conceptions unequivocally decline when social isolation increases. The effect is stronger for women who are between 21 and 25 years old and more educated, as well as for richer, larger, and more urban municipalities. COVID-19 is likely to change fertility across countries depending on the behavior of the population and on the lock-down measures implemented to fight the pandemic.

## Introduction

The Coronavirus disease 2019 (COVID-19) pandemic has significantly impacted several dimensions of human populations [1, 2]. While the main focus of media reports and scientific research has been on death tolls and vaccines, speculations have been made about possible fertility and birth changes during and after outbreaks. Some analysts have talked about a potential “baby boom” caused by the stay-at-home orders, which could lead couples to spend more time together and increase sexual activity [3]. Moreover, a report by the United Nations predicted that the decrease in the availability of family planning services and modern contraceptives could lead to millions of unintended pregnancies [4]. Others have argued that we should expect a “baby bust”, instead, as rising financial instabilities and overall uncertainty would contribute to couples abandoning or postponing pregnancy plans. Such a fall in pregnancies was observed during the 1918 influenza pandemic and the 2008 global financial crisis [5, 6].

The outlook is not clear, and it seems likely that there will be heterogeneous effects, both between and within countries. In high-income countries, where women generally have more control over their fertility, a reduction in work-life balance, followed by financial struggles caused by the economic downturn and restricted access to assisted reproductive technologies, may cause conceptions to decrease [7, 8]. Conversely, in medium and low-income countries, especially in rural areas, limited contraceptive methods and increases in poverty rates could lead to rising birth rates.

Other than socioeconomic disruptions caused by the pandemic, the virus itself may affect fertility. Studies have shown that COVID-19 infection may have temporary physiological impacts on both female and male fertility, affecting the menstrual cycle and semen quality [9–11] . Additionally, there were concerns about potential effects of vaccination on fertility, but recent evidence has shown there were no significant adverse effects [12, 13].

Early in the pandemic, evidence pointing towards a “baby bust” was seen in surveys showing revisions in pregnancy plans. Respondents reported postponing or abandoning previous intentions to conceive in European countries and in the United States [14, 15]. Yet, in another survey in the US, women reported having more difficulty accessing contraceptives, particularly among those most financially affected during the crisis, which could increase risks of unwanted pregnancies [16].

As preliminary data from the last quarter of 2020 became available, declines in crude birth rates were observed in rich countries [17, 18]. In Europe, this drop was associated with duration of lockdowns at the national level [19]. Evidence for medium and low-income countries, however, has been scarce. One study used data from telephone interviews in four countries in Africa, finding no indication of rising pregnancy rates during 2020 [20]. Another analyzed data from Moldova and found that there were reductions in pregnancy intentions and restrictions to some contraceptive methods, though these were largely offset by switches to other readily available methods [21]. Detailed analyses about possible causal mechanisms (financial instability vs fear of death vs lockdown related stress) have been sparse. One study analyzed a few counties in the United States and found that declines in birth rates were steeper where there were more infections of COVID-19 and more pronounced mobility reductions [22]. Another showed that social distancing policies in Japan were associated with reductions in pregnancies and areas with more rigorous precautions had a steeper decline [23]. Using an event study and difference-in-differences design with data on the United States and Europe, [24] found significant effects of lockdowns on Google searches for pregnancy-related term such as pregnancy tests and emergency contraception. Finally, a study using a difference-in-differences approach in Australia found that lockdowns had negative impacts on women’s fertility intentions [25]. We contribute to this literature by combining administrative microdata on all Brazilian births and fetal deaths, which allows us to calculate weekly conception numbers by municipality, with municipality-level daily geographical isolation data, to explore the impact of social isolation on the number of conceptions.

Regarding Brazil, previous literature studied the effect of the Zika epidemic in mid 2010s, showing that there was a decrease in conceptions probably caused by pregnancy postponement and increases in abortions [26]. These declines were steeper for more educated and younger women [27]. Still, there are significant differences between the Zika epidemic and the COVID-19 pandemic. Importantly, Zika was associated with microcephaly [28] so that one of the reasons for pregnancy postponing might have been fear of congenital malformations. This would not be a main factor in the current pandemic. In addition, the incidence and scale of the COVID-19 pandemic was much greater, with consequences spanning several socioeconomic areas. Implications for women’s health in particular have been extremely unequal across regions and income levels. Conceptions can increase if women’s control over their fertility choices are being reduced due to gender violence and mental health complications or if access to contraception is restricted [29]. Therefore, the extent and direction to which the pandemic might affect conceptions is unclear, and evidence for middle and low-income countries has been extremely limited.

In this context, this study aimed to investigate the association between staying at home during the pandemic and the number of conceptions in Brazil. We gathered data on all registered births in Brazil throughout 2020 and 2021, including date and place of birth, as well as mother’s characteristics. The database also informs gestational age at birth in weeks, so we can approximate the week of conception. To ensure our data consider the total number of conceptions and not only those leading to live births, we also collected data on the number of fetal deaths and estimate conception dates for pregnancies that did not lead to live births. We then combine these numbers with daily social isolation data by municipality, measured by an aggregate index of geographical isolation, and estimate econometric models to assess the effect of stay-at-home guidelines on conceptions.

## Materials and methods

Data wrangling and analysis were conducted with R version 4.1.2 [30], with the “tidyverse” [31], "estimatr” [32], “stargazer” [33], and “lfe” [34] packages. Moreover, calculations for margin effects were conducted with Stata version 15.0 [35] using package “reghdfe” [36].

### Data

We extracted birth data from the Sistema Nacional de Nascidos Vivos—SINASC, which is managed by the Brazilian Ministry of Health (specifically, by the Secretaria de Vigilância em Saúde SVS/MS). Data for all Brazilian states, from 2012 to 2020, were downloaded from DATASUS, a system used by the Ministry of Health to publicize data, and the preliminary 2021 data were from the SVS/MS website itself. Files were downloaded as DBC/DBF, which we converted to CSV for proper data cleaning and structuring. Each record represents a child born in Brazil, with detailed information about health at birth and mother characteristics. The variables we used in the analysis were municipality of residence, date of birth, mother’s education, gestational age at birth in weeks, the number of other children the mother has given birth to, and the mother’s age in years.

We then calculated the estimated date of conception for each birth, by subtracting seven times the number of gestation weeks from the date of birth. We deleted observations that had no data for birth weight, type of delivery, mother’s education or gestational length. We also excluded observations with mother age registered as less than 10 or more than 85 years old, which would almost certainly be input errors. All of those excluded observations accounted for ∼5% of total records. Additionally, we excluded births from three microregions (Fernando de Noronha, Traipu and Auriflama) which had too few records. Together, they account for less than 0.01% of the Brazilian population.

In our main statistical analysis, we control for the number of deaths in the municipality week. In order to do that, we downloaded data on deaths records in Brazil from the Sistema de Informações sobre Mortalidade (SIM), also managed by SVS/MS and published in DATASUS. We extracted data on municipality of residence and date of death for deaths in all Brazil from 2015 to 2020. Due to underreporting, excess mortality has typically been used to measure mortality in the pandemic, instead of confirmed COVID-19 deaths [37, 38]. In this context, [39] showed that excess mortality was negatively correlated with live birth numbers in Europe. We thus include total deaths and model weekly variations controlling for seasonal patterns.

Importantly, changes in birth patterns might be associated not with pregnancy rates, but with variability in fetal mortality and abortions. To account for this, we also obtained data on registered fetal deaths, also from SIM (DOFET—Declarações de Óbitos Fetais) and available in DATASUS. Again, as data for 2021 is preliminary, it was downloaded from a different source, the Portal Brasileiro de Dados Abertos–Base de Registros SIM 2021. We estimated the date of conception for those fetuses in the same way as we did with live births.

To measure social isolation, we used the Social Distancing Index calculated by In Loco, which leverages anonymized cellphone locational data from millions of devices across the country and calculates, by municipality and date, the share of people who stayed within a 450 radius of their houses. The index has been used extensively by the media and government throughout the pandemic, correlates adequately with other mobility indices such as Google Mobility and has also been used in several scientific publications [40, 41]. Other data sources included estimates of population in 2020 by municipality from the Instituto Brasileiro de Geografia e Estatística—IBGE and GDP per capita by municipality in 2018 (latest available data), also calculated by IBGE. To categorize municipalities into urban and rural, we again used IBGE’s classification.

Our study has an ecological design and uses only secondary data obtained from publicly available datasets, so it is exempt from approval by an institutional review board. Although the isolation index dataset uses anonymized individual location data to calculate the indices, it does not pose risks to individuals as it does not collect civil information such as name or social security number, and the data was aggregated by municipality by the company that collected it and publicized at the aggregated level. The birth microdata is also anonymized by the Brazilian Ministry of Health, and we aggregated it at municipality/week level, so that it is not possible to identify any individual-level information.

For our main dataset, data were grouped by municipality and week. We defined a week number as the number of weeks since December 30^th^, 2019. Week number 0, therefore, starts on Monday Dec 30^th^, 2019, and ends on Sunday Jan 5 ^th^ 2020; week number 1 starts on Monday Jan 6^th^ 2020 and ends on Sunday Jan 12 ^th^ 2020; etc. Our sample includes weeks 5 (starting on Monday, Feb 2^nd^) to 29 (starting on Monday, Jul 20^th^). Even though we have data for births throughout the whole year, our isolation data is limited for this time period (February to July) and therefore we are not able to analyze other months. Besides calculating total conceptions by municipality and week, we also calculated conceptions considering two categories of mother age and mother education: for age, we considered four categories corresponding to the quartiles of the age distribution in the sample; for education, we considered mothers who have not completed high school vs mothers who have completed high school or higher levels of education. This categorization was chosen based on the variables provided in the source datasets and in previous research that shows that completing high school is a strong determinant of fertility choices [42, 43]. We also grouped mothers in two categories according to the previous number of live children (no previous children vs one or more previous children.)

To group the isolation data, we first excluded municipalities for which there was no isolation data available for all days in our dataset (which encompasses February to July). The original dataset had data for 4778 municipalities (out of 5570 municipalities in Brazil), exclusion due to missing isolation data left us with 3633 and further exclusion of the three microregions mentioned previously left us with 3628 municipalities. Then, we calculated mean isolation by week for each municipality. Our main regression models, however, include only municipalities with more than ten births per week, to avoid abnormal variations in the change of log births. For robustness, we also created a dataset grouped by microregion instead of municipalities, as well as a dataset grouped by municipality and month instead of weeks to reduce measurement errors due to a very small number of births. A first look at the data also involved analyzing grouped data by state (Brazil has 27 states including the capital’s federal unit). To group by microregion and state, we weighted isolation data by each municipality’s population.

### Statistical analysis

We started with descriptive analysis, grouping the data by state and calculating changes in isolation between months to check whether these numbers were correlated with conceptions. We calculated changes in conceptions adjusting for seasonal effects by using double-differences, i.e., comparing changes in (log) conceptions over two consecutive months in 2020 with the same two months in 2019.

Many studies have applied statistical and mathematical models to different settings amid the COVID-19 pandemic. Transmission and mortality, for example, have typically been analyzed with SIR-Poisson or Bayesian modelling [44, 45]. The same approach has been used to model the effects of lockdowns and social distancing [46]. To assess causal effects, correcting for time-invariant factors in small units of analysis, high-dimensional fixed effect models have been widely used [47, 48]. Following this line of studies, in our main analysis, we aggregate all data by week and municipality and estimate Multi-Way Fixed-Effect models to assess the effect of social distancing on conceptions, starting with the following equation:

$$ln\;\left(Concep_{{}_{t,i}}\right)\;=\;\alpha_{i}\;+\beta \;Isolated_{t,i}\;+\;\gamma ln\left(Deaths_{t,i}\right)\;+\;\delta_{t}+\theta_{i}t\;+\;\varepsilon_{t,i},$$

Where *δ*_*t*_ are week fixed-effects that control for seasonal effects and the advance of the covid pandemic throughout Brazil, *α*_*i*_ are municipality fixed-effects that control for unobserved municipality-specific characteristics that are fixed over our sample period, such as population, GDP, location, poverty and other factors, and *Deaths*_*t*,*i*_ represents the number of deaths in the municipality/week, which could also play an important role in determining conceptions. The term *θ*_*i*_*t* allows for different municipality-specific time trends (t) over time and *ε*_*t*,*i*_ is a random error.

We take first-differences across successive weeks to eliminate the fixed effects, obtaining the equation that will be taken to the data:

$$\Delta ln\left(\;Concep{\;}_{t,i}\right)\;=\;\beta \Delta \;Isolated{\;}_{t,i}\;+\;\gamma \Delta ln\left(\;Deaths{\;}_{t,i}\right)\;+\;\delta_{t}\;+\;\theta_{i}\;+\;f_{im}\;+\;\Delta \varepsilon_{t,i},$$

In the main specification we also include interactions between municipality and months fixed-effects (*f*_*im*_), so that the variation used to estimate the effect of isolation comes solely across weeks of the same month, to control for seasonality effects in the change in conceptions.

Regressions are weighted by population size and are adjusted for clusters at the municipality levels to allow for heteroskedasticity and serial correlation within municipalities over time. We carry out several robustness tests, such as controlling for municipality-specific trends over time, aggregating the data to the microregion level and using months instead of weeks to allow for measurement errors in the computation of births. We compute heterogeneous effects by education, age, number of previous kids, municipality size and poverty levels (S1 and S2 Tables). We conduct placebo tests by regressing conceptions in each year between 2012 and 2019 on the 2020 week isolation rates to examine whether spurious correlations could be driving our results (S1 Fig). In the main analysis we exclude municipalities with less than 10 conceptions per week, but we also carry out robustness tests with different exclusion criteria, as well as using different model specifications, such as using absolute values instead of logs and estimating unweighted regressions (S3 Table). Finally, we also aggregate data by different weekdays to ensure our results are not driven by random chance due to our grouping criteria (S4 Table). All tests confirm the robustness of our results.

## Results

**Fig 1:**
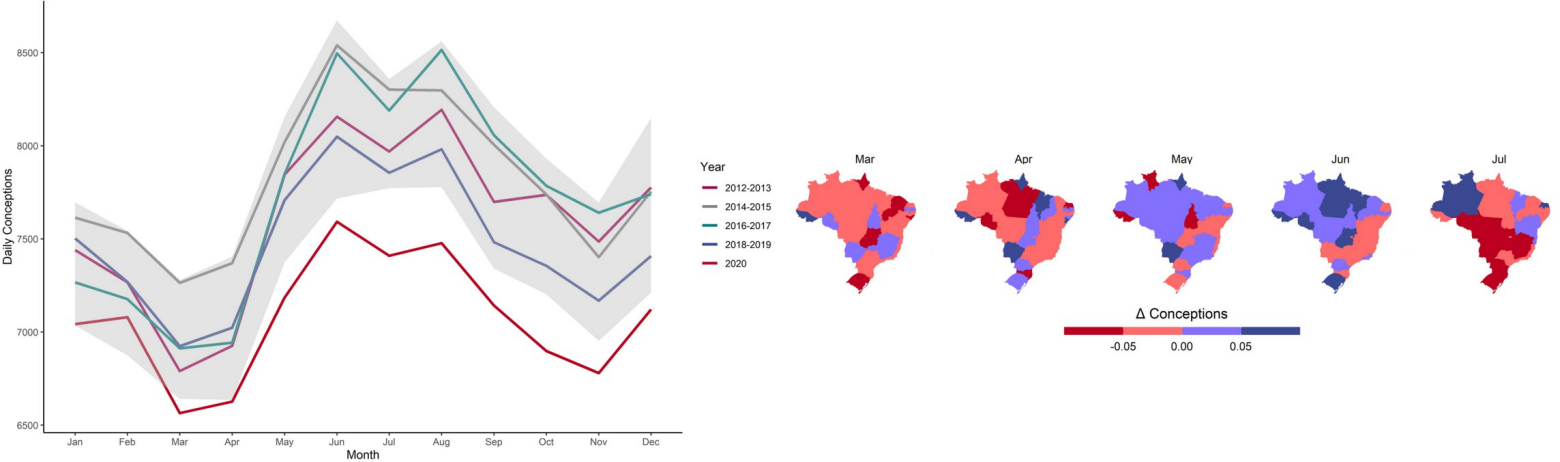
2020 conceptions in Brazil. (**A**) Total conceptions in Brazil by year. Conceptions are calculated as daily averages by month to account for different numbers of days in each month. Colored lines show two-year pairs averages (except for 2020) and the grey band shows minimum and maximum values from 2012 to 2019. (**B**) Double differences in conceptions by state in 2020. Rates are calculated as double differences in log conceptions, by subtracting conceptions in each month by the number in the previous month, and then taking the difference of this variation and the same variation in the previous year. This controls for seasonal changes that occur every year. The variation shown is thus calculated as: (*ln Concep*_*m*,2020_ –*ln Concep*_*m*-1,2020_)–*ln Concep*_*m*,2019_ –*ln Concep*_*m*-1,2019_) for *m* = March, April, May, June, and July.

Fig 1A shows that there is a clear decline in the total number of conceptions in 2020 relative to previous years, even after considering seasonal trends. Conceptions are calculated as daily averages by month to account for different numbers of days in each month. The colored lines show two-year pairs averages (except for 2020) and the grey band shows minimum and maximum values in each year from 2012 to 2019. Though fertility in Brazil had been declining prior to the pandemic [49], Fig 1A shows that the number of conceptions in January and February 2020 were still within the range of previous years. However, starting in March 2020, the number of conceptions dropped below the range of previous years for all months.

**Fig 2:**
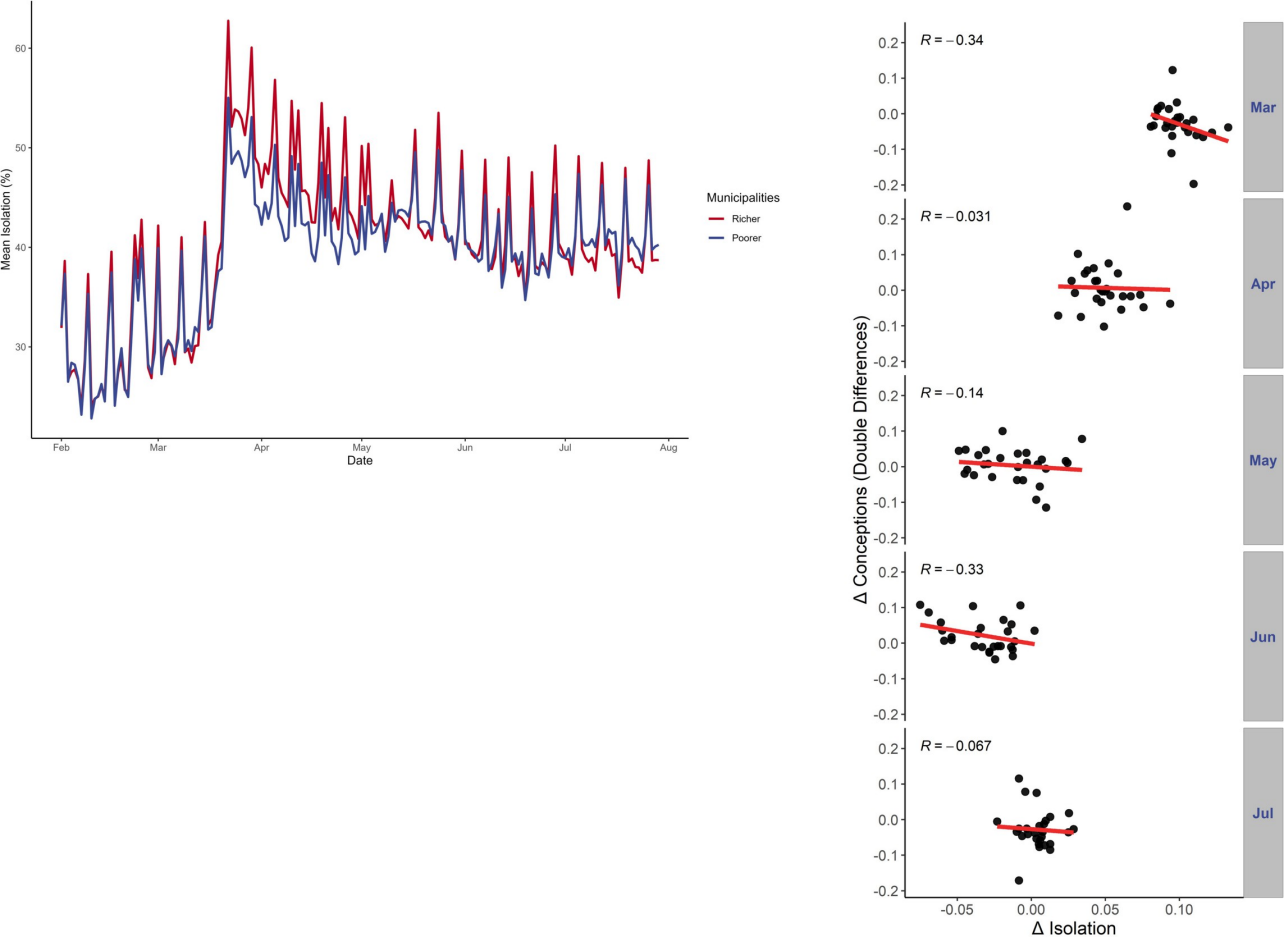
Isolation in Brazil. (**A**) Figure plots daily mean isolation index calculated as a weighted average of each municipality’s index, weighted by population. The index considers the share of people who stayed within a 450m radius to their houses on each day. Municipalities are grouped as rich or poor according to the median value of their GDP per capita, (**B**) Scatterplots of the monthly variation in isolation for each Brazilian state with respect to the previous month (X-axis) and double differences in conceptions, as defined in Fig 1B (Y-axis). *R* is the Pearson correlation coefficient.

Fig 1B shows changes in State-level conceptions rates adjusted for seasonal effects by using double-differences, i.e., comparing changes in (log) conceptions over two consecutive months in 2020 with the same two months in 2019. Monthly conceptions vary because human fertility is markedly seasonal, mainly due to physiological factors associated with temperature and photoperiod [50]. The figure shows that changes in conceptions vary substantially across States, with a marked decline in conceptions in April, especially in regions more affected by Covid-19 outbreaks, such as in the Amazon State, which saw a devastating outbreak in its capital (Manaus) in April [40]. Interestingly, the figure shows an increase in conceptions in these regions after May, suggesting a reversion back to the mean which coincides with a decrease in isolation rates (Fig 2A).

We then combined the data on conceptions with information on social isolation, measured by the share of people who stayed close to their houses, disaggregated by municipality and day. Fig 2A displays the behavior of isolation over time, comparing more educated with less educated individuals, showing that isolation increased more among the more educated. A simple correlation analysis with data aggregated by month and state displayed in Fig 2B shows a negative relationship between seasonally adjusted variation in conception rates and changes in isolation.

Table 1 reports the main results from our Fixed Effects models. Municipalities with less than ten births per week are excluded. Column (1) only controls for week fixed-effects, and the estimated coefficient is negative and statistically significant. Column (2) controls for the changes in the number of deaths, showing that mortality does not seem to impact the number of conceptions. Column (3) includes municipality fixed-effects to control for trends in conceptions over the months and the results do not change qualitatively. Column (4), our preferred specification, includes month fixed effects and municipality-month interactions, to capture variations within each month and municipality. The results are significant and even greater in magnitude. In column (5), we aggregated the data to the microregion level, as there are many municipalities with few conceptions in a week. The impact remains very similar. When examining the relationship at the municipality/month level in column (6), to allow for measurement errors, we again obtain similar results.

**Table 1:** Effect of isolation on conceptions.

	Δ ln Conceptions					
	(1)	(2)	(3)	(4)	(5)	(6)
Δ Isolation	-0.385***	-0.372***	-0.372***	-0.500***	-0.526***	-1.372***
	(0.133)	(0.133)	(0.133)	(0.166)	(0.141)	(0.052)
Δ ln Deaths		-0.007	-0.007	-0.007	0.0002	0.051***
		(0.008)	(0.008)	(0.009)	(0.009)	(0.014)
Two Way Fixed Effects:			Y	Y	Y	
Month interactions:				Y	Y	
Group by microregion:					Y	
Group by month:						Y
Observations	10,944	10,944	10,944	10,944	11,400	12,050
R^2^	0.032	0.032	0.034	0.101	0.119	0.125

Each regression (column) estimates the effect of social distancing on the number of conceptions. Columns (1)-(4) are grouped by week and municipality, column (5) is grouped by week and microregion and column (6) is grouped by month and municipality. Variables are included as first differences between successive weeks or months (Conceptions and Deaths are log-differences). All regressions are weighted by municipality or microregion population. Columns (1) and (2) include week fixed effects; columns (3) and (4) include week and municipality fixed effects; column (5) includes week and microregion fixed effects; column (6) includes municipality and microregion fixed effects. Columns (4) and (5) include municipality-month (or microregion-month) interactions and month fixed effects. Standard errors are reported in parentheses and clustered at the municipality or microregion level. Significance:

***p < 0.01

**p < 0.05

*p < 0.1.

**Fig 3:**
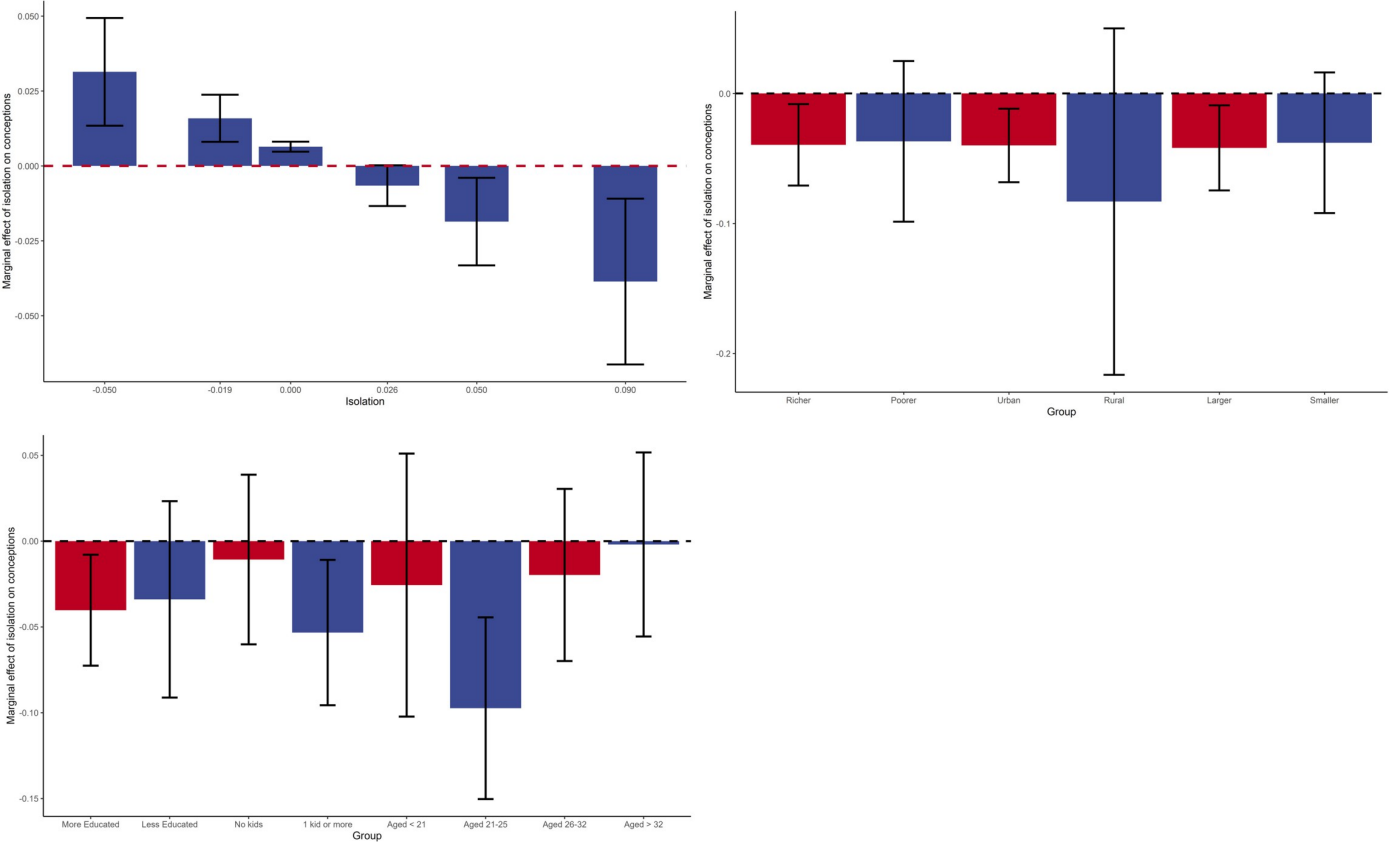
Marginal and heterogeneous effects of isolation on conceptions. The figure illustrates our main results based on Column (4) of Table 1, showing linear predictions of the effect of isolation on conceptions for the fitted model in our main specification (**A**) and heterogenous effects when considering different groups of either municipalities (**B**) or women (**C**). 95% C.I’s are also included. Shown in the heterogeneous effects plots are marginal effects for a 9 p.p change in isolation. Groups are defined as follows, with the total number of observations for each regression in parentheses: richer (poorer) municipalities—annual 2018 GDP per capita above (below) the median of BRL 17,427 (N = 8,352 and N = 2,592); urban/rural follows IBGE’s classification (N = 10,560 and N = 384); larger (smaller)—population above (below) 120,000 people (N = 6,336 and N = 4,608); more (less) educated are women who completed (did not complete) high school (N = 10,944 and N = 10,748); no kids vs 1 kid or more considers the number of previous births to live children the mother has given (N = 10,888 and N = 10,944); age categories are grouped according to the quartiles of the distribution of mothers in the sample (N = 10,587, N = 10,910, N = 10,937, and N = 10,777 respectively). Full regression results are shown in S1 and S2 Tables.

Fig 3A illustrates the main effects obtained in column (4), showing estimates and 95% confidence intervals (CI). It shows that when isolation decreases by 5 pp. the number of conceptions increases by 3.1% (CI 95% 1.3%-4.9%; effect size 0.10). An increase in isolation of 9 pp, on the other hand, decreases conceptions by 3.9% (CI 95% 6.6%-1.1%; effect size 0.18).

Fig 3B and 3C shed light on the different mechanisms that may be driving these results, by showing effects for different groups of municipalities and women’s characteristics of an increase in isolation of 9pp. When comparing richer and poorer municipalities, Fig 3B shows that the effect is only statistically significant in the richer ones, where the number of conceptions decreases by 3.9% (CI 95% 7.1–0.8%, effect size 0.18), in urban cities, with a similar decrease of 4.0% (CI 95% 6.8%-1.1%, effect size 0.18) and in bigger cities, decreasing by 4.2% (CI 95% 7.5%-0.9%, effect size 0.23). Meanwhile, the results are not significant for those municipalities that are smaller (CI 95% -9.2%–+1.6%), rural (CI 95% -21.6%–+5.0%), and poorer (CI 95% -9.9%–+2.5%),

Moreover, the more educated women (who completed high school) are most affected, with a decrease of 4.0% (CI 95% 7.3%-0.8%, effect size 0.16), whereas for less educated women the estimated effect is not statistically different from zero (CI 95% -9.1%–+2.3%). Women in the second quartile of the age distribution (aged between 21 and 25) are more affected, with a decrease in conceptions of 9.7% (CI 95% 15.0%-4.4%, , effect size 0.20) when the share of people staying close to their homes increased by 9pp. Meanwhile, the effect is not statistically significant among women in the other age groups (aged < 21 : CI 95% -10.2%–+5.1%; aged 26–32 : CI 95% -7.0%–+3.0%; aged > 32 : CI 95% -5.6%–+5.2%;). Finally, the effect is slightly larger for women who had already given birth to live children previously at 5.3% (CI 95% 9.6%-1.1%, effect size 0.18) while it is not significant for women who did not have previous children (CI 95% -6.0%–+3.9%).

## Discussion

Our results show that there is a robust negative relationship between the share of people isolated and the number of conceptions during the COVID-19 pandemic in Brazil. The effect is stronger for younger and more educated women, as well as for richer, larger, and more urbanized municipalities. This result is in line with what was observed during the Zika epidemic in Brazil [26, 27], and with theories about potential heterogeneous effects of isolation on pregnancy behavior amid the COVID-19 pandemic.

It has been argued that a fertility decline would be more likely in high income regions due to a worsening of the work-life balance, rising financial struggles, and reduced access to assisted reproductive technologies. Additionally, the lack of outsourced childcare imposes more burden on parents since children are always at home, which could also lower conceptions rates [7]. In poorer areas, the impacts could go in the opposite direction, as restricted access to contraceptive methods would lead to increases in unintended pregnancies, and rising inequality and poverty levels could represent setbacks in the long term reductions in fertility that have been associated with development in the past decades [7]. Teenage pregnancies could rise in these areas as well, due to prolonged school closures [51]. Although we are not able to pin down the exact reason behind the fertility changes during stay-at-home periods, our findings support these theoretical predictions, and shed light on what the main drivers of these observations might be.

First, it seems that most potential increases in fertility during the pandemic would be due to unintended pregnancies. Indeed, in the UK unintended pregnancies almost doubled in 2020, mainly due to restricted access to contraception [52]. The heterogeneities we observe also support this conclusion, as we find that the fertility reductions were larger for more educated women. It has been also argued that economic shocks are more likely to affect younger women’s pregnancy decisions, as they have more fertile time left to make up for postponed childbearing [53]. Therefore, the group most likely to reduce planned pregnancies during the social isolation period would be those women who still have some fertile years remaining but are not in their teenage years, which are more prone to unintended pregnancies. In line with these predictions, the largest effect was observed among women aged 21 to 25 years old, representing the second quartile of the age distribution in our sample. This suggests that a significant portion of the decline in conceptions witnessed in Brazil is likely attributable to planned pregnancies.

The decision to postpone childbearing during the pandemic could also be caused by fear of infection. Mothers might fear getting ill during pregnancy and have to go to crowded hospitals, where the risk of contamination is higher, or that the newborn might contract the virus right after labor. Although there have been reports of such concerns in surveys with pregnant women, they have also responded being worried about changes in maternity services, such as availability of midwifes and restrictions on partners’ attendance at birth [54, 55]. Since the number of weekly deaths in each municipality is not significant in our statistical models, it seems that fear of contracting the virus itself is not a main factor in fertility changes. Instead, the heterogeneities we observe suggest that the main reason driving this reduction is the increase in overall uncertainty and financial instability leading to postponement of pregnancies.

Rising unemployment is associated with fertility reductions, but financial concerns are not the only reason why shutdowns could reduce pregnancy intentions [56]. The psychological burden of isolation increases stress and anxiety which can negatively impact fertility choices [57]. The weekly granularity of our data could suggest that the immediate financial consequences of shutdowns are not as significant as overall stress and concerns regarding the potential duration and extension of the lockdown measures. That is, weekly changes in isolation patterns have more immediate consequences in anxiety and stress, whereas unemployment might not fluctuate as much due to fixed costs and labor contracts [58]. Moreover, educated women have access to more information during the pandemic and thus might be more susceptible to such fears and stress, as opposed to financial concerns, especially since they are more likely to be able to work from home without losing any income. Therefore, our findings suggest that the main drivers of the reduction in fertility are increases in stress and overall uncertainty that lead young, educated mothers to postpone planned pregnancies.

Interestingly, [25] found opposite results in Australia: lockdowns had negative impacts on fertility intentions, but these effects were more pronounced among older and less educated women. This may be explained for several reasons, such as large differences between the two countries, particularly considering that there were stronger lockdown policies in Australia. Moreover, the outcome they measured is fertility plans, not actual births. Especially considering that Brazil’s unequal reality has impacts on women health and fertility [59], an increase in unwanted pregnancies among less educated women in Brazil could have offset any reductions in planned pregnancies. This shows that regions that have distinct socioeconomic and cultural profiles had different effects on fertility, which points to a need for further research.

It remains an open question, however, whether these reductions will have a long-term impact on fertility changes in Brazil and worldwide. Since the number of conceptions increase again when isolation decreases, these effects alone are unlikely to cause long-term fertility changes in Brazil. Yet, social distancing was only one of the many ways in which the pandemic affected society, which could also have impacted fertility. Moreover, it is possible that this was more pronounced during the first wave in the beginning of 2020, when fear and uncertainty was highest, and might not have been as intense in following outbreaks. Still, even as vaccination advanced early in 2021, Brazil suffered a severe third wave of COVID- 19 and the Brazilian Health Ministry asked women, particularly younger ones, to postpone pregnancies when possible [60].

One potential limitation of our study is sampling bias, since we do not have data on every municipality in the country. However, we include data for all 27 Brazilian states, which have very different sociodemographic characteristics, and we test several sampling strategies to alleviate this concern. In our main specification, we exclude municipalities in which there are weeks with 0 deaths or less than 10 conceptions. Therefore, our main sample represents 456 municipalities (and we have data for 24 weeks, thus N = 10,944 in Table 1), which account for about 62% of the total Brazilian population. When we group by microregion, we keep 475 microregions (out of 558 in the country), accounting for over 95% of the country’s population. Finally, when grouping by municipality and month, we keep 2,410 municipalities (times 5 months, N = 12,050), accounting for ∼88% of the population. To ensure our results are not driven by these arbitrary criteria, we carry out robustness tests with different exclusion criteria and different model specifications, and all tests confirm our results.

Additionally, our recent data are preliminary, which could also bias results. However, the Ministry of Health continuously receives birth information from State and municipality governments throughout the year and updates its databases. Although the microdata are only consolidated and released twice a year, there is a system that continuously keeps track of the total number of registered births as they are updated [61]. Comparing our preliminary data with the most recent numbers posted on this webpage, there is no difference for all months in 2020, and differences smaller than 2%, 3% and 7% for the first three months of 2021 respectively. This could, however, still cause small differences in our results if the missing data are not randomly distributed across municipalities and weeks. A related potential problem is that there might also be measurement errors in the reported gestation weeks which we use to estimate conception dates, as well as in other reported information such as mother’s age and education. The robustness tests that we carry out by grouping observations by microregion and month help alleviate these concerns regarding measurement error and outdated data.

External validity might be another concern, both spatially and temporally. We only use data that refer to the first months on the pandemic, and Brazil might have specific cultural characteristics that lead to unique fertility patterns. Further studies should address cross-country heterogeneous effects to study implications for population trends worldwide, as well as continue investigating these effects to assess whether any future waves will have different consequences.

## Conclusions

We combined administrative data on births and fetal deaths with daily geographical isolation data to assess the effects of social distancing on the number of conceptions in Brazil. Using high dimensional fixed effects models, we found a robust negative relationship between the share of people isolated and the number of conceptions during the COVID-19 pandemic in Brazil. The effect is stronger for women between 21 and 25 years old and more educated, as well as for richer, larger, and more urbanized municipalities.

The main drivers of the reduction in fertility appear to be increases in stress and overall uncertainty that lead young, educated mothers to postpone planned pregnancies. However, detailed analyses of the mechanisms are needed. Future research could explore different study designs that can provide robust evidence regarding the causal mechanisms behind the relationship between isolation and fertility, such as the role of stress, anxiety, and uncertainty in shaping pregnancy decisions. Moreover, it remains an open question whether these reductions will have a long-term impact on fertility changes in Brazil and worldwide, and whether other aspects of the COVID-19 pandemic also affected fertility.

Our findings have important implications for policymakers and healthcare providers. It highlights the need to take fertility into account in situations not immediately related to reproductive health. This relates to the need to improve access to contraception and reproductive health services, especially in poorer areas where restricted access to contraceptive methods can lead to increases in unintended pregnancies.

## Supporting information

S1 Fig Placebo tests for several years. Figure shows coefficients and 95% CI’s for the effect of social isolation on conceptions when using conception data for different years (placebo regressions). All regressions follow the main specification of column (4) of Table 1, such that the last data point on the chart (2020) represents our main specification. Variables are included as first differences between successive weeks (Conceptions and Deaths are log-differences). All regressions are weighted by municipality population and standard errors are clustered at the municipality level.
(TIF)

S1 Table Heterogeneous effects of isolation on conceptions for different municipality groups. Each regression (column) estimates the effect of social distancing on the number of conceptions in a subsample of municipalities. Variables are included as first differences between successive weeks or months (Conceptions and Deaths are log-differences). Groups are defined as follows: richer (poorer) municipalities—annual 2018 GDP per capita above (below) BRL 17,427; urban/rural follows IBGE’s classification; larger (smaller)—population above (below) 120,000 people. All regressions are weighted by municipality population and include month, week and municipality fixed effects and municipality-month interactions. Standard errors are reported in parentheses and clustered at the municipality level. Significance: ***p < 0.01; **p < 0.05, *p < 0.1.
(DOCX)

S2 Table Heterogeneous effects of isolation on conceptions for different women groups. Each regression (column) estimates the effect of social distancing on the number of conceptions for a group of women. Variables are included as first differences between successive weeks or months (Conceptions and Deaths are log-differences). Groups are defined as follows: more (less) educated are women who completed (did not complete) high school; no kids vs previous kids considers the number of previous births to live children the mother has given; age groups are divided according to the quantiles in the sample. All regressions are weighted by municipality population and include month, week and municipality fixed effects and municipality-month interactions. Standard errors are reported in parentheses and clustered at the municipality level. The number of observations reported might not match the numbers mentioned in the caption for Fig 3 due to the exclusion of singleton observations for the margin calculations. Significance: ***p < 0.01; **p < 0.05, *p < 0.1.
(DOCX)

S3 Table Alternative model specifications. Each regression (column) estimates the effect of social distancing on the number of conceptions. Variables are included as first differences between successive weeks (Conceptions and Deaths are log-differences, except in column (4)). Columns (1)-(4) exclude municipalities with 0 deaths or less than 10 conceptions per week. Column (5) includes all municipalities for which we have data. Column (6) excludes municipalities with 0 deaths or conceptions per week. Column (7) excludes municipalities with 0 deaths or less than 20 conceptions per week. Weighted regressions are weighted by municipality population. Standard errors are reported in parentheses and clustered at the municipality level. Significance: ***p < 0.01; **p < 0.05, *p < 0.1.
(DOCX)

S4 Table Aggregation by different weekdays. Each regression (column) estimates the effect of social distancing on the number of conceptions aggregating daily data by different weekdays. Column (1) is the baseline specification, and aggregates data weekly starting on Monday Feb 3rd 2020. Column (2) aggregates data starting on Tuesday Feb 4th 2020, Column (3) on Wednesday Feb 5th 2020 etc. Variables are included as first differences between successive weeks (Conceptions and Deaths are log-differences). Regressions are weighted by municipality population and include month, week and municipality fixed effects and municipality-month interactions. Standard errors are reported in parentheses and clustered at the municipality level. Significance: ***p < 0.01; **p < 0.05, *p < 0.1.
(DOCX)
