# Supplementary figures and images for: The relationship between staying at home during the pandemic and the number of conceptions: A national panel data analysis
Source: PLoS One. 2023 Aug 11;18(8):e0289604. doi: 10.1371/journal.pone.0289604 (PMC10420359; doi:10.1371/journal.pone.0289604)

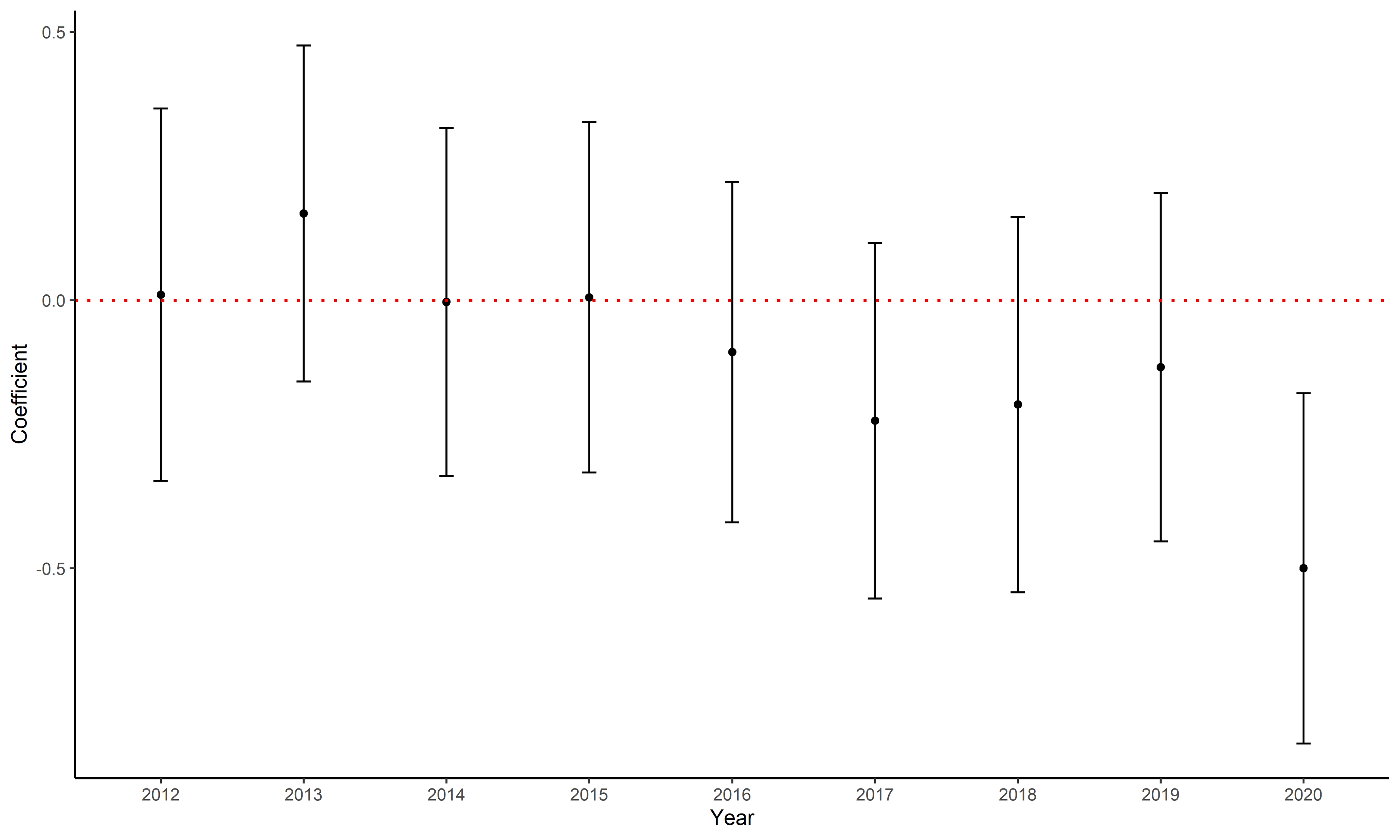

Supplement: S1 Fig — Figure shows coefficients and 95% CI’s for the effect of social isolation on conceptions when using conception data for different years (placebo regressions). All regressions follow the main specification of column (4) of Table 1, such that the last data point on the chart (2020) represents our main specification. Variables are included as first differences between successive weeks (Conceptions and Deaths are log-differences). All regressions are weighted by municipality population and standard errors are clustered at the municipality level. (TIF) [file pone.0289604.s001.tif]
