# Supplementary material for: The relationship between staying at home during the pandemic and the number of conceptions: A national panel data analysis
Source: PLoS One. 2023 Aug 11;18(8):e0289604. doi: 10.1371/journal.pone.0289604 (PMC10420359; doi:10.1371/journal.pone.0289604)
Supplement: S1 Table — Each regression (column) estimates the effect of social distancing on the number of conceptions in a subsample of municipalities. Variables are included as first differences between successive weeks or months (Conceptions and Deaths are log-differences). Groups are defined as follows: richer (poorer) municipalities—annual 2018 GDP per capita above (below) BRL 17,427; urban/rural follows IBGE’s classification; larger (smaller)—population above (below) 120,000 people. All regressions are weighted by municipality population and include month, week and municipality fixed effects and municipality-month interactions. Standard errors are reported in parentheses and clustered at the municipality level. Significance: ***p < 0.01; **p < 0.05, *p < 0.1. (DOCX) [file pone.0289604.s002.docx]

**S1 Table. Heterogeneous effects of isolation on conceptions for different municipality groups.**

|  | | | | | | |
| --- | --- | --- | --- | --- | --- | --- |
|  | Δ ln Conceptions | | | | | |
|  |  | | | | | |
|  | Poorer | Richer | Larger | Smaller | Urban | Rural |
|  | (1) | (2) | (3) | (4) | (5) | (6) |
|  | | | | | | |
| Δ Isolation | -0.498 | -0.508*** | -0.539*** | -0.491 | -0.516*** | -1.037 |
|  | (0.370) | (0.188) | (0.196) | (0.322) | (0.170) | (0.776) |
|  |  |  |  |  |  |  |
| Δ ln Deaths | -0.040** | 0.004 | -0.011 | -0.003 | -0.005 | -0.052 |
|  | (0.017) | (0.010) | (0.013) | (0.012) | (0.009) | (0.034) |
|  |  |  |  |  |  |  |
|  | | | | | | |
| Observations | 2,592 | 8,352 | 6,336 | 4,608 | 10,560 | 384 |
| R^2^ | 0.108 | 0.103 | 0.110 | 0.089 | 0.101 | 0.183 |
|  |  |  |  |  |  |  |

Each regression (column) estimates the effect of social distancing on the number of conceptions in a subsample of municipalities. Variables are included as first differences between successive weeks or months (Conceptions and Deaths are log-differences). Groups are defined as follows: richer (poorer) municipalities - annual 2018 GDP per capita above (below) BRL 17,427; urban/rural follows IBGE’s classification; larger (smaller) - population above (below) 120,000 people. All regressions are weighted by municipality population and include month, week and municipality fixed effects and municipality-month interactions. Standard errors are reported in parentheses and clustered at the municipality level. Significance: ***p < 0.01; **p < 0.05, *p < 0.1.

1
