# Supplementary material for: The relationship between staying at home during the pandemic and the number of conceptions: A national panel data analysis
Source: PLoS One. 2023 Aug 11;18(8):e0289604. doi: 10.1371/journal.pone.0289604 (PMC10420359; doi:10.1371/journal.pone.0289604)
Supplement: S2 Table — Each regression (column) estimates the effect of social distancing on the number of conceptions for a group of women. Variables are included as first differences between successive weeks or months (Conceptions and Deaths are log-differences). Groups are defined as follows: more (less) educated are women who completed (did not complete) high school; no kids vs previous kids considers the number of previous births to live children the mother has given; age groups are divided according to the quantiles in the sample. All regressions are weighted by municipality population and include month, week and municipality fixed effects and municipality-month interactions. Standard errors are reported in parentheses and clustered at the municipality level. The number of observations reported might not match the numbers mentioned in the caption for Fig 3 due to the exclusion of singleton observations for the margin calculations. Significance: ***p < 0.01; **p < 0.05, *p < 0.1. (DOCX) [file pone.0289604.s003.docx]

| **S2 Table. Heterogeneous effects of isolation on conceptions for different women groups.** | | | | | | | | |
| --- | --- | --- | --- | --- | --- | --- | --- | --- |
|  | | | | | | | | |
|  | Δ ln Conceptions | | | | | | | |
|  |  | | | | | | | |
|  | Less Educ | More Educ | Previous kids | No kids | Aged < 21 | Aged 21-25 | Aged 26-32 | Aged > 32 |
|  | (1) | (2) | (3) | (4) | (5) | (6) | (7) | (8) |
|  | | | | | | | | |
| Δ Isolation | -0.435 | -0.521^***^ | -0.670^***^ | -0.172 | -0.332 | -1.196^***^ | -0.281 | -0.074 |
|  | (0.344) | (0.194) | (0.248) | (0.293) | (0.460) | (0.318) | (0.301) | (0.322) |
|  |  |  |  |  |  |  |  |  |
| Δ ln Deaths | 0.015 | -0.013 | -0.008 | -0.014 | 0.003 | -0.014 | -0.024 | 0.014 |
|  | (0.020) | (0.010) | (0.011) | (0.013) | (0.020) | (0.018) | (0.015) | (0.017) |
|  |  |  |  |  |  |  |  |  |
|  | | | | | | | | |
| Observations | 10,779 | 10,944 | 10,944 | 10,894 | 10,619 | 10,912 | 10,937 | 10,781 |
| R^2^ | 0.078 | 0.095 | 0.087 | 0.088 | 0.087 | 0.074 | 0.079 | 0.084 |
|  | | | | | | | | |
| Note: | ^*^p^**^p^***^p<0.01 | | | | | | | |

Each regression (column) estimates the effect of social distancing on the number of conceptions for a group of women. Variables are included as first differences between successive weeks or months (Conceptions and Deaths are log-differences). Groups are defined as follows: more (less) educated are women who completed (did not complete) high school; no kids vs previous kids considers the number of previous births to live children the mother has given; age groups are divided according to the quantiles in the sample . All regressions are weighted by municipality population and include month, week and municipality fixed effects and municipality-month interactions. Standard errors are reported in parentheses and clustered at the municipality level. The number of observations reported might not match the numbers mentioned in the caption for Figure 3 due to the exclusion of singleton observations for the margin calculations. Significance: ***p < 0.01; **p < 0.05, *p < 0.1.
