# Supplementary material for: The relationship between staying at home during the pandemic and the number of conceptions: A national panel data analysis
Source: PLoS One. 2023 Aug 11;18(8):e0289604. doi: 10.1371/journal.pone.0289604 (PMC10420359; doi:10.1371/journal.pone.0289604)
Supplement: S3 Table — Each regression (column) estimates the effect of social distancing on the number of conceptions. Variables are included as first differences between successive weeks (Conceptions and Deaths are log-differences, except in column (4)). Columns (1)-(4) exclude municipalities with 0 deaths or less than 10 conceptions per week. Column (5) includes all municipalities for which we have data. Column (6) excludes municipalities with 0 deaths or conceptions per week. Column (7) excludes municipalities with 0 deaths or less than 20 conceptions per week. Weighted regressions are weighted by municipality population. Standard errors are reported in parentheses and clustered at the municipality level. Significance: ***p < 0.01; **p < 0.05, *p < 0.1. (DOCX) [file pone.0289604.s004.docx]

**S3 Table. Alternative model specifications.**

|  | Δ ln Conceptions | | | | | | | | | |
| --- | --- | --- | --- | --- | --- | --- | --- | --- | --- | --- |
|  |  | | | | | | | | | |
|  | Baseline |  |  | No log | No filter | | Filter 0 | | Filter 20 | |
|  | (1) | (2) | (3) | (4) | (5) | | (6) | | (7) | |
|  | | | | | | | | | | |
| Δ Isolation | -0.500*** | -0.507** | -0.310* | -0.151 | | -0.393*** | | -0.339** | | -0.599*** |
|  | (0.166) | (0.215) | (0.178) | (0.117) | | (0.118) | | (0.143) | | (0.190) |
|  |  |  |  |  | |  | |  | |  |
| Δ ln Deaths | -0.007 | -0.008 | -0.008 |  | | 0.003 | | 0.001 | | -0.012 |
|  | (0.009) | (0.009) | (0.009) |  | | (0.004) | | (0.006) | | (0.012) |
|  |  |  |  |  | |  | |  | |  |
| Δ Deaths |  |  |  | -0.011 | |  | |  | |  |
|  |  |  |  | (0.007) | |  | |  | |  |
|  |  |  |  |  | |  | |  | |  |
|  | | | | | | | | | | |
| Weighted: | Y |  |  | Y | | Y | | Y | | Y |
| Month dummies: | Y | Y |  | Y | | Y | | Y | | Y |
| Observations | 10,944 | 10,944 | 10,944 | 10,944 | | 60,826 | | 28,296 | | 6,048 |
| R^2^ | 0.101 | 0.089 | 0.023 | 0.994 | | 0.125 | | 0.082 | | 0.114 |
|  | | | | | | | | | | |

Each regression (column) estimates the effect of social distancing on the number of conceptions. Variables are included as first differences between successive weeks (Conceptions and Deaths are log-differences, except in column (4)). Columns (1)-(4) exclude municipalities with 0 deaths or less than 10 conceptions per week. Column (5) includes all municipalities for which we have data. Column (6) excludes municipalities with 0 deaths or conceptions per week. Column (7) excludes municipalities with 0 deaths or less than 20 conceptions per week. Weighted regressions are weighted by municipality population. Standard errors are reported in parentheses and clustered at the municipality level. Significance: ***p < 0.01; **p < 0.05, *p < 0.1.
