# Supplementary material for: The relationship between staying at home during the pandemic and the number of conceptions: A national panel data analysis
Source: PLoS One. 2023 Aug 11;18(8):e0289604. doi: 10.1371/journal.pone.0289604 (PMC10420359; doi:10.1371/journal.pone.0289604)
Supplement: S4 Table — Each regression (column) estimates the effect of social distancing on the number of conceptions aggregating daily data by different weekdays. Column (1) is the baseline specification, and aggregates data weekly starting on Monday Feb 3rd 2020. Column (2) aggregates data starting on Tuesday Feb 4th 2020, Column (3) on Wednesday Feb 5th 2020 etc. Variables are included as first differences between successive weeks (Conceptions and Deaths are log-differences). Regressions are weighted by municipality population and include month, week and municipality fixed effects and municipality-month interactions. Standard errors are reported in parentheses and clustered at the municipality level. Significance: ***p < 0.01; **p < 0.05, *p < 0.1. (DOCX) [file pone.0289604.s005.docx]

**S4 Table. Aggregation by different weekdays.**

|  | Δ ln Conceptions | | | | | | |
| --- | --- | --- | --- | --- | --- | --- | --- |
|  | Monday | Tuesday | Wednesday | Thursday | Friday | Saturday | Sunday |
|  | (1) | (2) | (3) | (4) | (5) | (6) | (7) |
|  | | | | | | | |
| Δ Isolation | -0.500*** | -0.603*** | -0.454** | -0.328* | -0.401** | -0.390** | -0.413*** |
|  | (0.166) | (0.168) | (0.180) | (0.187) | (0.168) | (0.154) | (0.156) |
|  |  |  |  |  |  |  |  |
| Δ ln Deaths | -0.007 | -0.012 | -0.009 | -0.002 | -0.001 | -0.009 | -0.004 |
|  | (0.009) | (0.008) | (0.008) | (0.008) | (0.008) | (0.008) | (0.008) |
|  |  |  |  |  |  |  |  |
|  | | | | | | | |
| Observations | 10,944 | 11,016 | 10,872 | 10,920 | 10,968 | 10,968 | 11,016 |
| R^2^ | 0.101 | 0.101 | 0.103 | 0.117 | 0.109 | 0.107 | 0.100 |
|  | | | | | | | |

Each regression (column) estimates the effect of social distancing on the number of conceptions aggregating daily data by different weekdays. Column (1) is the baseline specification, and aggregates data weekly starting on Monday Feb 3rd 2020. Column (2) aggregates data starting on Tuesday Feb 4th 2020, Column (3) on Wednesday Feb 5th 2020 etc. Variables are included as first differences between successive weeks (Conceptions and Deaths are log-differences). Regressions are weighted by municipality population and include month, week and municipality fixed effects and municipality-month interactions. Standard errors are reported in parentheses and clustered at the municipality level. Significance: ***p < 0.01; **p < 0.05, *p < 0.1.
